# Supplementary figures and images for: A computational model for bacteriophage ϕX174 gene expression
Source: PLoS One. 2024 Oct 31;19(10):e0313039. doi: 10.1371/journal.pone.0313039 (PMC11527146; doi:10.1371/journal.pone.0313039)

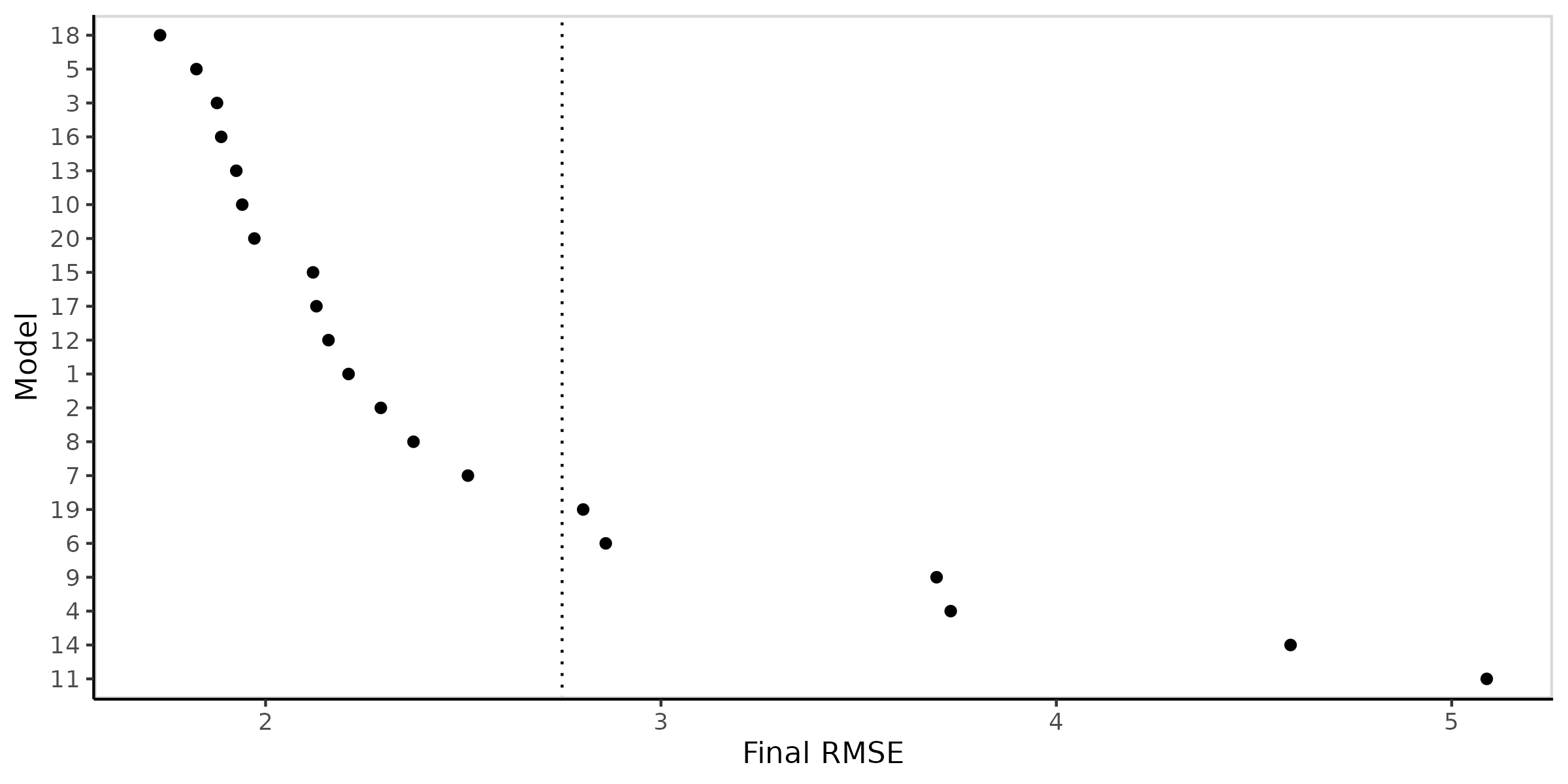

Supplement: S1 Fig — Each point corresponds to the simulation that had the best (lowest) RMSE over the 4000 training generations. The dashed line marks the manually-defined cutoff score used in downstream analysis. (PNG) [file pone.0313039.s002.png]

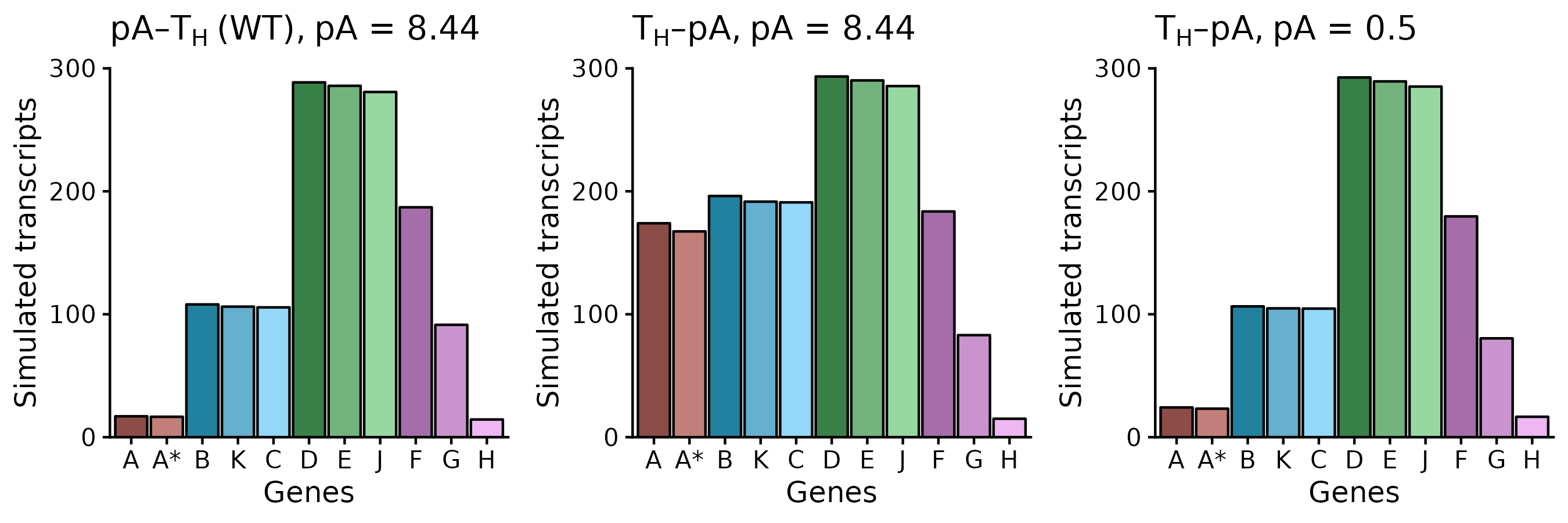

Supplement: S2 Fig — Left panel: simulation of wild-type ϕX174 (pA before TH) with parameter values obtained from fitting simulations to qPCR data. Middle panel: simulation with pA/TH order reversed (TH before pA) and the same pA binding strength as the fitted simulation (pA = 8.44). Transcript abundances for genes A/A* are about 10 times greater than wild-type, and abundances for genes B/K/C are about 2 times greater than wild-type. Right panel: simulation with pA/TH order reversed (TH before pA) and a pA binding strength of 0.5. The value of 0.5 was obtained by manually adjusting promoter A to revert transcript abundances for genes A/A*/B/K/C back to their wild-type ratios. For all panels, simulated transcript abundances are steady-state quantities averaged over five replicate simulations. (PNG) [file pone.0313039.s003.png]

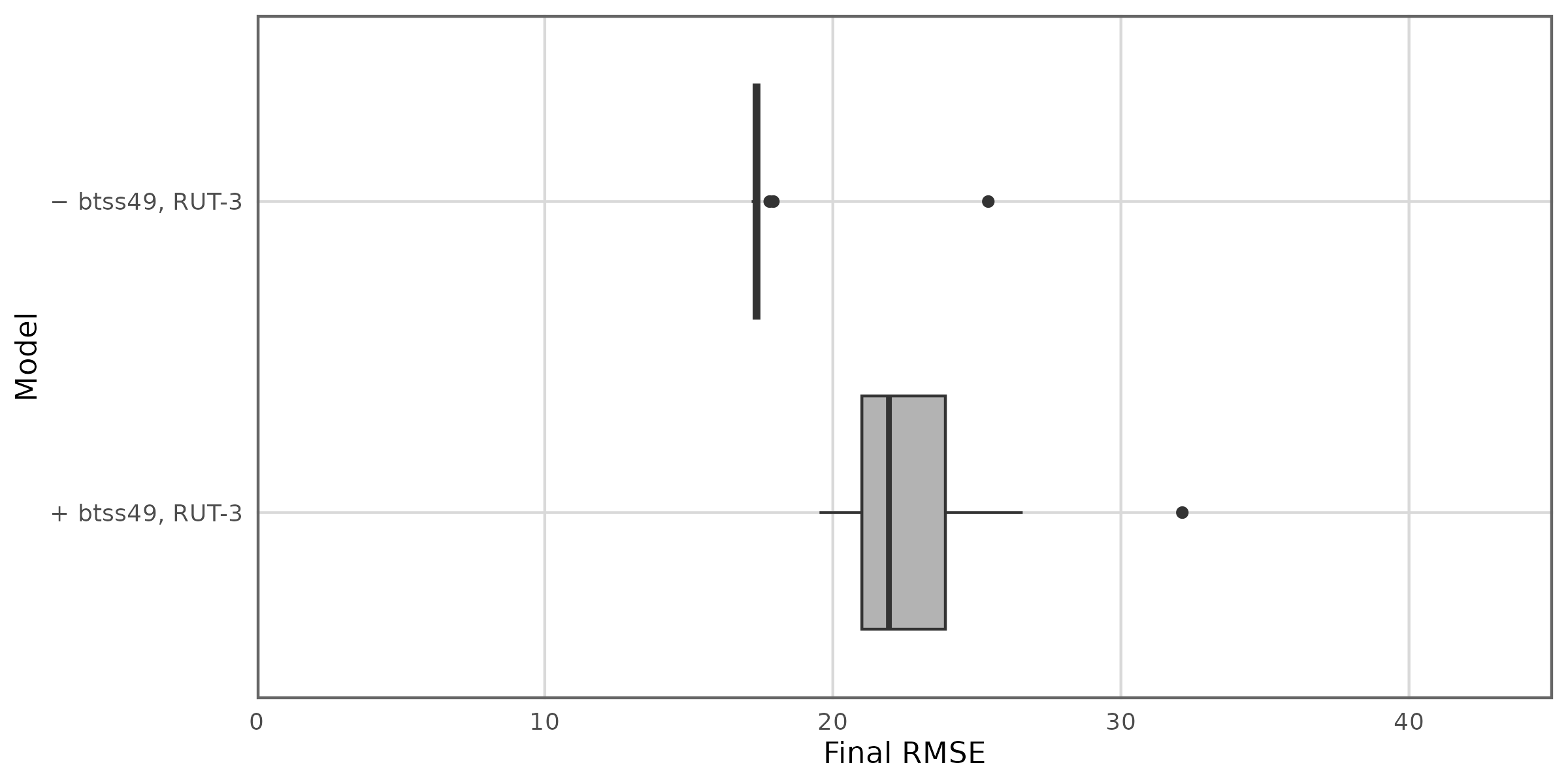

Supplement: S3 Fig — Each point corresponds to one simulation that had the best (lowest) RMSE over the 4000 training generations. − btss49, RUT-3: Simulations with the canonical ϕX174 regulatory model. + btss49, RUT-3: Simulations with an alternative regulatory model proposed by Logel and Jaschke [12], with one additional promoter and one additional terminator. The mean RMSE for simulations with the alternative model is significantly higher than the mean for simulations with the canonical model only (p < 0.05, Student’s t-test). (PNG) [file pone.0313039.s004.png]

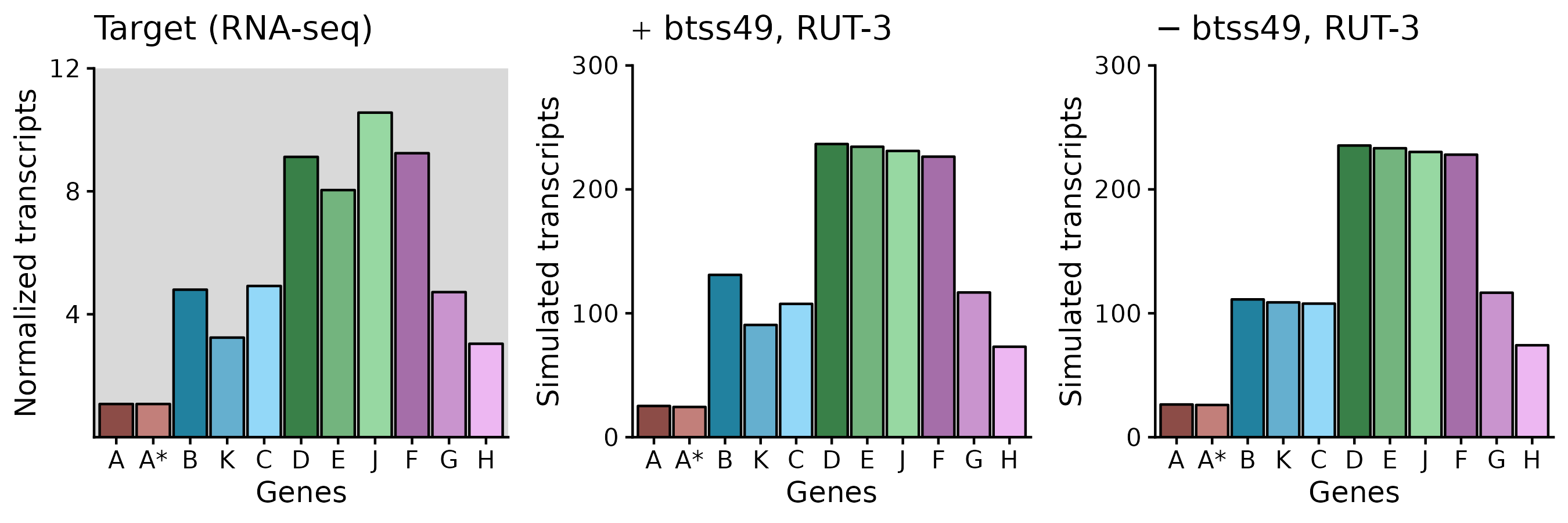

Supplement: S4 Fig — Left panel: Target ϕX174 transcription data, measured using RNA-seq. Transcript abundances shown are per million reads, normalized to gene A. The transcription data was collected by Logel and Jaschke [12]. Middle panel: Output from the best fit simulation (simulation with the lowest RMSE out of 20 independently fit models) with putative promoter btss49 and putative terminator RUT-3. Right panel: Output from the best fit simulation of the canonical regulatory model. (PNG) [file pone.0313039.s005.png]

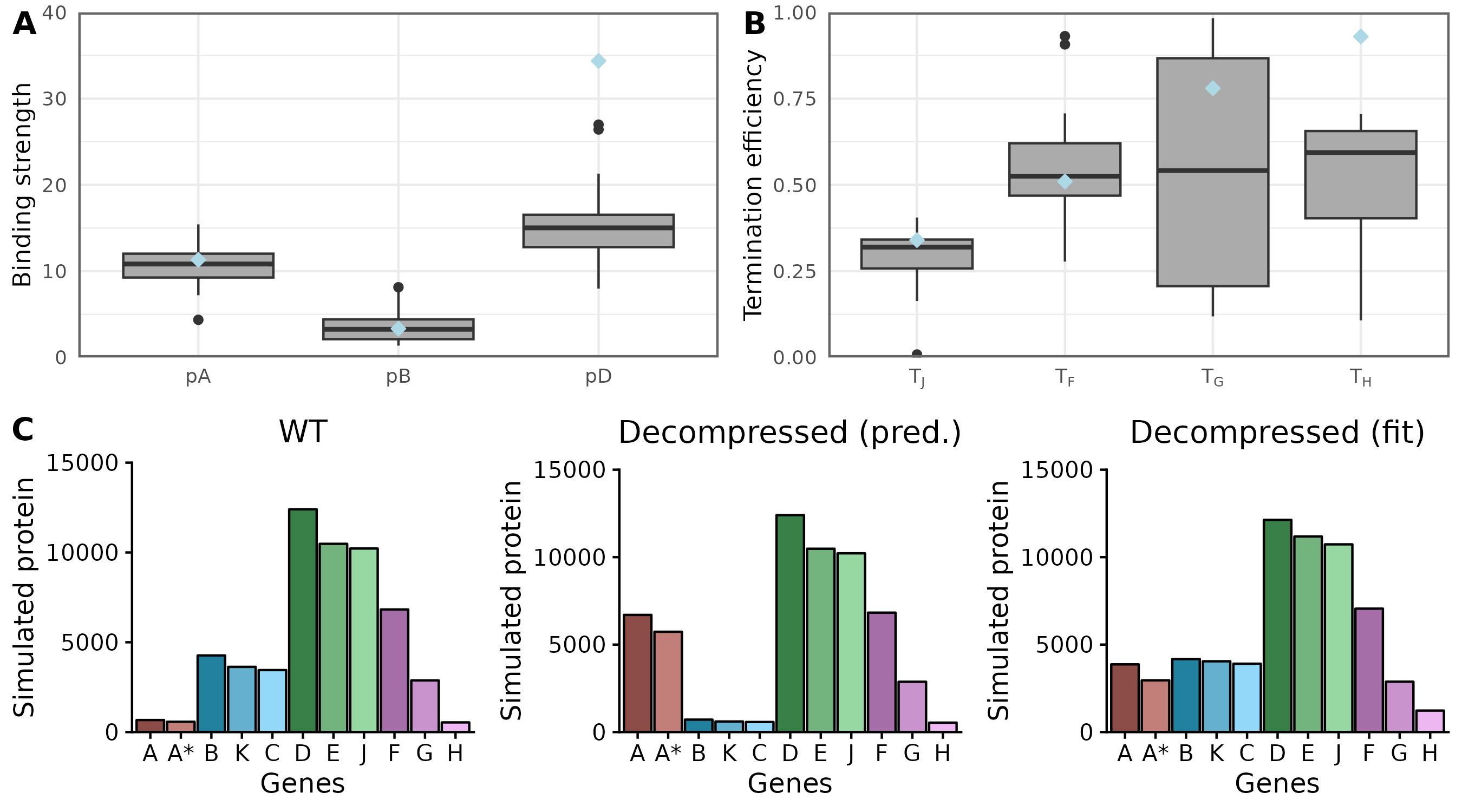

Supplement: S5 Fig — The target protein abundance was prepared by adjusting simulated wild-type protein abundances to match fold-change differences as reported by Wright et al. [6] (see also Materials and methods). Promoter distributions (A) and terminator distributions (B) from 20 independently fit models. In both A and B, the light blue diamonds are the mean parameter estimates from fitting the wild-type model to qPCR data. C: Final simulated protein abundances from the decompressed model. Simulated wild-type protein abundances (left panel) and the target, adjusted protein abundances (middle panel) are shown for reference. (PNG) [file pone.0313039.s006.png]
