# Supplementary material for: A computational model for bacteriophage ϕX174 gene expression
Source: PLoS One. 2024 Oct 31;19(10):e0313039. doi: 10.1371/journal.pone.0313039 (PMC11527146; doi:10.1371/journal.pone.0313039)
Supplement: Attachment — Submitted filename: Reviewer_response.pdf [file pone.0313039.s007.pdf]

Response to the Reviewer Comments

We would like to thank both reviewers for their careful reading of our manuscript and for their helpful comments. We have made every effort to address the comments in the revised manuscript.

Reviewer 1

The manuscript "A computational model for bacteriophage φX174 gene expression" talks about the construction of a computational model for φX174 and using the model to study gene regulation during the phage infection cycle. They build a model for bacteriophage φX174 gene expression using a customizable stochastic simulation framework that was previously used to model bacteriophage T7 infection dynamics.They modeled the phage genome as a linear molecule with a start site 63 nucleotides upstream of gene A. To simulate circular genome transcription, polymerases that reach the end of the genome without terminating automatically begin another round of transcription starting from the beginning of the genome. They estimated the strengths of promoters and terminators by fitting the simulations to φX174 transcription data. They first fit the model to qPCR data of φX174 and then they fit the simulations to higher-resolution RNA-seq data. For the canonical model (without putative regulatory elements), they found that the fit to RNA-seq data is similar but not exactly identical to the fit to qPCR data. The φX174 genome was previously refactored to remove all gene overlaps a process referred to as genome decompression. In the decompressed φX174 variant, all partially or completely overlapping coding regions were copied and placed side-by-side. The decompressed φX174 strain was viable but showed reduced fitness compared to wild-type φX174, although the exact causes of the fitness reduction were unclear. The authors conclude by saying that their work demonstrates that φX174 gene regulation is well described by the canonical set of promoters and terminators widely used in the literature. The authors also emphasize, that the approach here was not to develop a one-off, purpose-built models that can describe only one particular organism or biological system, as commonly seen in the field of biological simulation. Instead, they leveraged the Pinetree simulator, which is a general-purpose prokaryotic gene expression simulator that can be adapted relatively easily to any system of interest.

This manuscript is well written. The methods are explained in detail and the conclusions are based on the results. The manuscript though did not provide any new knowledge to the field but provides a framework for future studies.

Response: We respectfully disagree that our manuscript does not provide any new knowledge. Our manuscript provides evidence that the two new proposed regulatory elements are likely not real, or are at least very weak. Our manuscript also provides evidence that the observed reduced phiX174 fitness after genome decompression likely cannot be explained by changes to transcription alone.

Reviewer 2

This research article presents an impressive and detailed computational simulation of ϕX174 gene expression, offering significant insights into the bacteriophage's transcriptional regulation. The use of mechanistic modeling to explore decompression and regulatory element behavior is well-executed, and the comparison between wild-type and decompressed models contributes valuable knowledge to the field of viral gene regulation. The use of two distinct data sources (qPCR and RNA-seq) strengthens the robustness of the findings.

Positive Aspects:

The study uses sophisticated computational simulations to understand viral gene regulation, which is a valuable contribution to virology research.
The comparison between wild-type and decompressed ϕX174 genomes offers new insights into the effect of gene rearrangement on transcription and expression.
The use of both qPCR and RNA-seq data adds depth to the findings, providing a comparative analysis of different regulatory models.
Suggestions for Minor Corrections:

Grammar and Spelling:

Line 185: Change "may also have disrupted" to "may have also disrupted."
We have made the suggested change to the manuscript.

Line 203: "We have used this model" should be "We used this model" for consistency with past tense.
In the first paragraph of the discussion (beginning on line 203), we use present perfect to indicate that this paragraph is a summary of the completed study. We have verified that tenses are used consistently throughout this portion of the discussion.

Line 216: "engineering the decompressed genome had effects" could be improved to "engineering of the decompressed genome affected."
We have made the suggested change to the manuscript.

Line 254: "the expression level of a given gene is often the aggregate result" should be "often results from the aggregate."
We have revised the sentence (modified text in bold):

> A priori, such effects may be caused by packaging defects if the enlarged genome no longer fits into the phage particle, by disrupted gene regulation—**as the expression level of a given gene often results from the aggregate activity of multiple, staggered promoters**—, or by the accidental disruption of unknown ORFs or regulatory elements during the engineering efforts.

Technical Consistency:

The reasoning for why a substantial decrease in TG and TH activities is unlikely should be elaborated for clarity, as it jumps to a conclusion without adequate evidence.
Response: Since tG and tH are both outside the overlapping gene region, it is unlikely that they were directly affected by the decompression process, which refactored portions of the genome between gene A* and gene J. It is possible, however, that a decrease in G/H expression could occur due to a mechanism not captured by our model.

In the Results, the subsection on simulating decompressed PhiX174, we have added the following text to clarify this:

> It is unlikely that TG and TH activities were directly affected by the decompression process, since they are outside of the region that was refactored. Although it is possible that decompression had an indirect effect on G and H expression, due to, for example, complex interplay between transcription and translation de-regulation, our fit of transcription elements only could not have captured such an effect.

In the Results section, the explanation of model fitting for decompressed vs. wild-type ϕX174 is detailed but could benefit from a clearer breakdown of specific observations with more explanation on fitting difficulties (such as TG variability).
Response: The lack of model convergence when allowing only promoters and terminators to change suggests to us that additional mechanisms are likely responsible for the decrease in phiX174 fitness. We have made the following changes to the manuscript to emphasize this (modified text in bold):

> **In addition, the estimate for tG did not converge fully, unlike in the wildtype model.** It is also impossible for the model to achieve a simultaneous increase in protein A/A* expression and decrease in B/C expression by only adjusting promoter and terminator strengths (S5C Fig). **Overall, we were unable to achieve a good fit to the decompressed phenotype by only allowing promoters and terminators to vary.** This result points to a cause other than gene rearrangements or accidental disruptions to promoter/terminator sequences for the altered protein expression phenotype observed for decompressed ϕX174.

Flow and Clarity:

The overall flow between sections can be improved. In particular, the transition between the discussion of decompression and transcription regulation feels abrupt. More context on decompression's impact on transcription could help bridge these ideas.
Response: We have added the following text at the beginning of the subsection “Simulating decompressed ϕX174” in Results:

> In addition to promoter and terminator arrangements, another important aspect of ϕX174 genome organization is the presence of multiple overlapping genes. In a prior study, the ϕX174 genome was refactored to separate all primary coding sequences, in order to better understand the regulatory consequences of gene overlaps (Jaschke et al. 2012).

We have also reviewed the entire manuscript for overall flow and clarity, and have made minor edits in particular in the Introduction, but also in Results and Discussion, to help smooth transitions between sections and ideas. For ease of review, we have provided a version of the manuscript with all changes highlighted.
